# Characterization of the Interaction of Human γS Crystallin with Metal Ions and Its Effect on Protein Aggregation

**DOI:** 10.3390/biom14121644

**Published:** 2024-12-21

**Authors:** Reinier Cardenas, Arline Fernandez-Silva, Vanesa Ramirez-Bello, Carlos Amero

**Affiliations:** 1 LABRMN, Centro de Investigaciones Químicas, Instituto de Investigación en Ciencias Básicas y Aplicadas, Universidad Autónoma del Estado de Morelos, Cuernavaca 62209, Mexico; reinier.cardenazmen@uaem.edu.mx (R.C.); arline.fernandezsil@uaem.edu.mx (A.F.-S.); vanesa.ramirezbel@uaem.edu.mx (V.R.-B.); 2 Grupo de Investigación en Producción y Sanidad en Ciencias Veterinarias y Zootecnias (PROSAVEZ), Facultad de Medicina Veterinaria y Zootecnia, Fundación Universitaria San Martín, Cali 760001, Colombia

**Keywords:** crystallins, human gamma crystallins, copper, zinc, cataracts

## Abstract

Cataracts are diseases characterized by the opacity of the ocular lens and the subsequent deterioration of vision. Metal ions are one of the factors that have been reported to induce crystallin aggregation. For HγS crystallin, several equivalent ratios of Cu(II) promote protein aggregation. However, reports on zinc are contradictory. To characterize the process of metal ion binding and subsequent HγS crystallin aggregation, we performed dynamic light scattering, turbidimetry, isothermal titration calorimetry, fluorescence, and nuclear magnetic resonance experiments. The data show that both metal ions have multiple binding sites and promote aggregation. Zinc interacts mainly with the N-terminal domain, inducing small conformational changes, while copper interacts with both domains and induces unfolding, exposing the tryptophan residues to the solvent. Our work provides insight into the mechanisms of metal-induced aggregation at one of the lowest doses that appreciably promote aggregation over time.

## 1. Introduction

Cataracts remain one of the leading causes of blindness worldwide [1] and are defined as the opacity that clouds the ocular lens, causing vision impairment. This opacity is due to the presence of protein aggregates in the lens [2,3]. Among the proteins that aggregate are crystallins, which constitute 90% of the proteins present in the lens [4], and the other 10% correspond to ion channels, proteins associated with metabolism, and cytoskeletal proteins that contribute to maintaining the shape of the lens [5].

**Figure 1:**
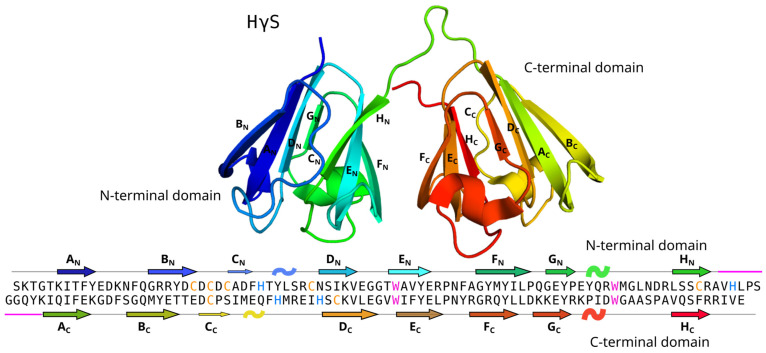
HγS crystallin structure and sequence. The three-dimensional structure of HγS crystallin is shown (PDB id 2M3T), highlighting the N-terminal domain and the C-terminal domain. The sequence is shown at the bottom, with cysteines marked in yellow, histidines in blue, and tryptophans in magenta.

Crystallins are grouped into two families: α-crystallins and βγ-crystallins [6]. α-Crystallins are small heat shock proteins that act as chaperones that bind partially unfolded proteins, sequestering them to prevent protein aggregation [6,7]. βγ-Crystallins are structural proteins composed of two β-sheet domains, each with two Greek-key motifs [8,9,10,11]. In adult lenses, one of the most abundant γ-crystallins is human gamma S crystallin (HγS crystallin), which has 178 residues, of which 7 are cysteines, and 4 are histidines [12,13,14] (Figure 1).

The crystallins are arranged such that the transparency of the lens is ensured [6,15]. As lenses age, crystallins accumulate damage over time, which could modify their primary structure, reduce their stability, and cause protein aggregation [3,16]. Among the external risk factors associated with cataract formation is metal ion exposure [16,17]. The concentration of metal ions in the healthy lens has been reported to be in the range of 0.4 and 30 µg metal/g lens tissue [18,19]; however, the concentrations of copper and zinc in lenses with cataracts increase [20,21].

Several studies have shown that metal ions, mostly copper and zinc, can induce aggregation in gamma [22,23] and beta crystallins [17,24]. This aggregation is concentration-dependent, with multiple binding sites and different aggregation mechanisms.

Specifically, for HγS crystallin, it was found that one equivalent ratio of Cu(II) does not induce appreciable aggregation [25,26], whereas additional equivalents promote unfolding and different aggregation pathways depending on the dose [26]. The evidence is less clear for Zn(II), with one report stating that this metal ion does not induce any aggregation [27], whereas a later report shows aggregation with more than one equivalent ratio [28].

These studies have shed light on metal–protein interactions mostly at high concentrations (up to 10 equivalent ratios); however, cataract formation due to exposure to metal ions is probably a slow, accumulative, low-dose process in which small, local damage caused by the metal ion induces protein aggregation over time. We report here the effects of copper and zinc at the lowest doses, which appreciably promote aggregation over time. Turbidimetry and dynamic light scattering (DLS) were used to track the formation of large light-scattering aggregates, whereas fluorescence and isothermal titration calorimetry (ITC) revealed features of interaction and aggregation due to the metals. Additionally, to provide insight into the interactions between HγS crystallin and the metal ions, nuclear magnetic resonance (NMR) perturbations were used to identify which residues of the protein are relevant for the interaction and aggregation.

## 2. Materials and Methods

All reagents were used without further purification and were obtained from Merck & Co., Inc., Kenilworth, NJ, USA. CuSO_4_ and ZnSO_4_ were used as sources of Cu(II) and Zn(II) ions, respectively.

### 2.1. Expression and Purification of HγS Crystallin

The recombinant protein was overexpressed in *E. coli* BL21-RIL cells grown at 37 °C in Super Broth medium supplemented with 100 μg/mL ampicillin and 30 μg/mL chloramphenicol. The production of HγS crystallin-SUMO was induced by the addition of 0.5 mM isopropyl β-d-thiogalactoside (IPTG) when the cells reached an OD600 of 2.0, and the cells were harvested after 18 h of incubation at 25 °C by centrifugation. The 15 N-labeled protein was grown at 37 °C in minimal M9 media supplemented with 1 g/L 15N-ammonium chloride as the sole nitrogen source. The cells were resuspended in 50 mM Tris and 10 mM imidazole, pH 8.0 (buffer A), and lysed by sonication.

The protein was purified using a three-step protocol. In the first step, chromatography was performed using a 5 mL HisTrap™ affinity column (Cytiva, Marlborough, MA, USA) charged with 100 mM nickel sulfate and equilibrated with buffer A. Second, the C-terminal tags were removed by incubation at 25 °C with SUMO protease Ulp1 for 12 h. Finally, the sample was passed through a second 5 mL HisTrap™ affinity column (Cytiva, Marlborough, MA, USA) to remove the His-tagged SUMO protein and protease. Protein purity and integrity were assessed by SDS-PAGE (Figure S7). The protein concentration was estimated from the predicted extinction coefficient of 41,200 M cm at 280 nm.

### 2.2. Turbidimetry

Measurements were made using an Agilent 8453 UV–visible diode array spectrophotometer (Agilent, Santa Clara, CA, USA) at 405 nm every 15 s. The protein concentration was 50 μM. Changes in turbidity over time were measured by incubating the proteins in the absence or presence of 1.5 equivalent ratios of CuSO_4_ or ZnSO_4_ at 37 °C. The measurement variation was estimated from three successive recorded data points, and the measurement error was calculated. The experiments were carried out in triplicate for each condition.

### 2.3. Dynamic Light Scattering

Data were acquired using a Zetasizer Nano ZSP spectrophotometer (Malvern Panalytical, Egham, UK) with a dispersion angle of 173°. Proteins were prepared at 50 μM. All the samples were centrifuged for 30 min at 10,000 rpm and filtered through a 0.22 μm filter before measurement. Changes in size over time were measured by incubating the proteins in the absence or presence of 1.5 equivalent ratios of CuSO_4_ or ZnSO_4_ at 37 °C. Triplicate experiments were performed for each condition. The data were analyzed via distribution methods.

The data were used to obtain translational diffusion coefficients through measurement of the correlation coefficient. The hydrodynamic radius, R_H_, was obtained from the diffusion coefficient, D, via the Stokes–Einstein equation. The R_H_ distribution is estimated, assuming that the molecules are spheres. The data were analyzed using the Zetasizer Software v8.02 (Malvern Panalytical, Egham, UK). Predicted hydrodynamic radii were calculated using Hullrad server [29].

### 2.4. Fluorescence

Fluorescence spectra were recorded on a Cary Eclipse spectrophotometer (Agilent, Santa Clara, CA, USA) using 1 cm light path cuvettes. Spectra were collected at a protein concentration of 50 μM at 37 °C. Conformational changes over time of the proteins in the absence and presence of 1.5 equivalent ratios of CuSO_4_ or ZnSO_4_ were followed by tryptophan (Trp) fluorescence at 37 °C. The emission spectrum was recorded in the range of 300–500 nm using an excitation wavelength of 295 nm. The maximum signal was used to estimate the change in the Trp environment and protein unfolding. Triplicate experiments were performed for each condition.

To determine the Fmax value, fluorescence spectra were recorded within the range of 300 to 500 nm, with excitation at 295 nm. The data were subsequently fitted to a Lorentzian function.

### 2.5. Isothermal Titration Calorimetry

ITC measurements were performed via a MicroCal ITC200 (Malvern Instruments, Northampton, MA, USA). The titration was carried out at 37 °C with stirring at 750 rpm. A total of 19 injections were performed, each adding 2 μL of 0.825 mM CuSO_4_ or ZnSO_4_ solution to a 55 μM protein sample.

### 2.6. Nuclear Magnetic Resonance

The experiments were performed at 37 °C on a JEOL JNM-ECZR 600 MHz spectrometer (JEOL Ltd., Akishima, Japan). A series of two-dimensional ^1^H-^15^N Heteronuclear Single Quantum Coherence (HSQC) experiments were acquired in the absence and presence of CuSO_4_ or ZnSO_4_ with 10 scans and 64 points in the indirect dimension.

The backbone resonance assignments at 37 °C were transferred from previous assignments deposited in the BioMagResBank (accession numbers 6253 and 27170) [30,31]. All the spectra were processed with NMRPipe v11.7 [32] and analyzed using CARA v1.9.1.8a2 [33]. The NMR samples were 500 μM HγS crystallin in 50 mM Tris, pH 8.0. The signal intensities and positions were measured for each spectrum and analyzed independently. The signal intensities were internally normalized with respect to a well-resolved peak to compensate for signal loss due to the formation of large aggregates induced by the metals. An error estimate of 10% was added to the peak intensity.

## 3. Results

Cataract formation is a slow accumulative process, with metal ion exposure being one of the risk factors involved in the development of this pathology. Previous reports have shown that high doses of Cu(II) induce HγS crystallin aggregation [25,26]; however, there are reports with different results for Zn(II) [27,28].

### 3.1. Cu(II) and Zn(II) Induce HγS Crystallin Aggregation

To study the mechanism of aggregation induced by low metal ion doses over time and considering that it has been reported that no significant aggregation occurs with 1 equivalent ratio or sub-stoichiometric concentration of the metal ions, 1.5 equivalent ratios of Cu(II) and Zn(II) were selected as the minimum concentration to observe aggregation at 37 °C.

We investigated the oligomeric state of HγS crystallin by dynamic light scattering (DLS) and used turbidimetry assays to monitor the formation of large aggregates over time. It should be noted that DLS is an extremely sensitive technique for the detection of large particles in solution, even at low concentrations. Therefore, even if the sample remains mostly monomeric, we should be able to detect the metal-induced formation of large aggregates, while turbidity assays will indicate whether the formation of these aggregates scatters light over time.

**Figure 2:**
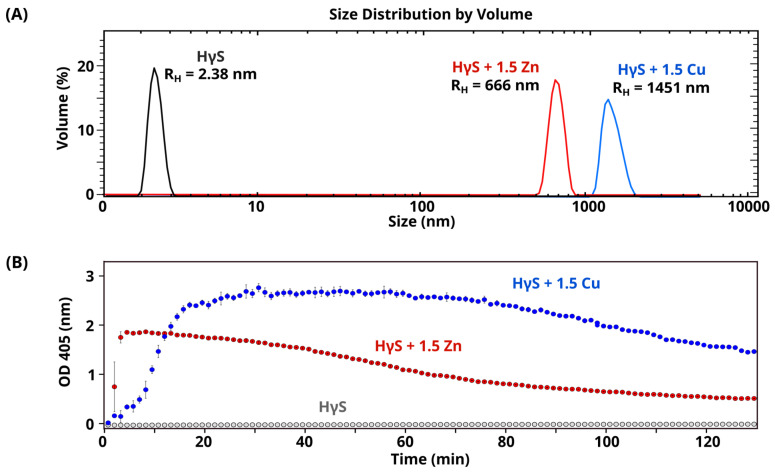
Effect of metal ions on the aggregation of HγS crystallin. (**A**) DLS size distribution diagram by volume of HγS crystallin (black), HγS + Zn(II) (red), and HγS + Cu(II) (blue). A shift to the right can be observed, indicating the formation of large aggregates. (**B**) Turbidity assays of HγS crystallin in the absence (gray) or presence of Cu(II) (blue) and Zn(II) (red).

Using the coordinates derived from the crystal structure of HγS crystallin [14], the translational diffusion coefficient (D_t_) predicted by hydrodynamic calculations was 1.04 × 10^−6^ cm^2^/s, which corresponds to an R_H_ ≈ 2.2 nm (Hullrad server [29]). DLS measurements of solutions containing HγS crystallin protein were polydisperse, with an apparent diffusion coefficient of 1.3 × 10^−6^ cm^2^/s for the major contribution. Therefore, under these conditions, HγS crystallin corresponded to proteins mainly in the monomeric state (R_H_ ≈ 2.3 nm) (Figure 2 and Figure S1). In the absence of metal ions, the HγS crystallin solution at 37 °C remained transparent over time (Figure 2B).

The samples were subsequently analyzed in the presence of metal ions to determine whether a change in protein size could induce turbidity upon metal addition. In the presence of 1.5 equivalent ratios of Cu(II), HγS crystallin forms large aggregates, as shown by the DLS correlation curve shifting to the right (Figure S1B). These aggregates reach large sizes (Figure 2A). Surprisingly, the interaction of HγS crystallin with 1.5 equivalent ratios of Zn(II) immediately led to the formation of large aggregates as soon as the metal was added (Figure 2B). With zinc, the aggregates reached sizes greater than 600 nm in R_H_ (Figure 2A). For both metals, large aggregates induce an increase in the absorbance detected at 405 nm (Figure 2B) due to the formation of large particles that scatter light over time, followed by a decrease in the OD observed over longer times. This decrease is a result of the formation of large aggregates that scatter less light [34] and some protein precipitation. The formation of aggregates in the presence of Cu(II) reached higher values, which were consistent with the measurements by DLS.

These data confirm that both copper and zinc induce the formation of large aggregates that scatter light even at low doses. Interestingly, despite similar results, small differences are observed, suggesting different mechanisms in the aggregation pathways for each metal.

### 3.2. Cu(II) and Zn(II) Induce Conformational Changes in HγS Crystallin

**Figure 3:**
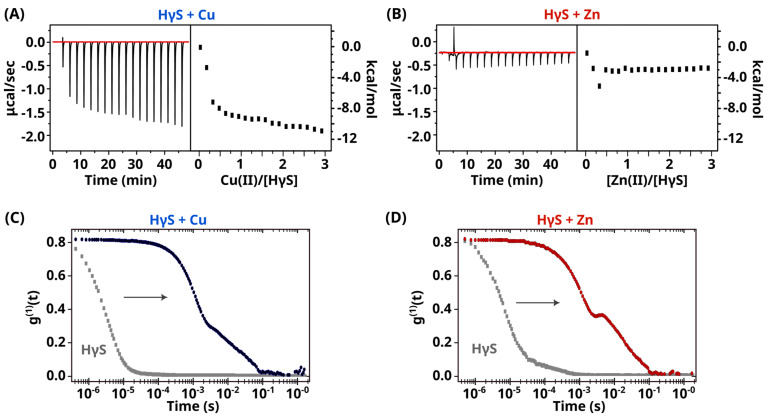
Metal binding by ITC. (**A**) Isothermal titration calorimetry of HγS crystallin bound to Cu(II). (**B**) Isothermal titration calorimetry of HγS crystallin bound to Zn(II). The left side shows the experimental isothermal titrations, whereas the right side shows the reaction heat. Both metals exhibited complex behavior involving several processes accounting for the heat reactions. (**C**) DLS correlograms of HγS crystallin in the absence (gray) and presence of Cu(II) (blue) after ITC titration. A shift to the right (indicated with an arrow) can be observed, indicating the formation of large aggregates. (**D**) DLS correlograms of HγS crystallin in the absence (gray) and presence of Zn(II) (red) after ITC titration. A shift to the right (indicated with an arrow) can be observed, indicating the formation of large aggregates.

We then decided to use ITC to obtain information on the binding of HγS crystallin to Cu(II) and Zn(II). As shown in Figure 3A, the addition of Cu(II) produces an unmistakable heat change in the sample. However, we observed an increase in peak area as we titrated the metal rather than the normal decrease. This behavior suggests that, even at low equimolar ratios, simple binding does not occur. Instead, metal binding probably induces additional events, such as unfolding and aggregation.

Surprisingly, for Zn(II), we did not detect any appreciable heat change. However, the DLS data for the samples before and after metal titration confirmed that Zn(II) induced the formation of large aggregates, indicating interactions with the protein. A possible explanation for these results is that Zn(II) also induces several events with opposing heat signatures, resulting in a net zero change in the heat detected in the ITC experiments (Figure 3B and Figure S2).

To evaluate whether Cu(II) and Zn(II) ions promote conformational changes in addition to aggregation, which could help to understand the thermodynamic signature, we used Trp fluorescence. The change in the chemical environment of the four buried tryptophan residues and their exposure to the solvent results in a rightward shift of the emission maximum. This shift has been used as an indicator of protein unfolding. To measure the maxima changes in the presence of the metals, thermal unfolding experiments were performed (Figure S3). The maximum fluorescence emission of the folded protein was 325 nm, whereas when the protein was unfolded, it shifted to 350 nm. A ratio of 350/325 was used to provide information about the folding state of the protein.

**Figure 4:**
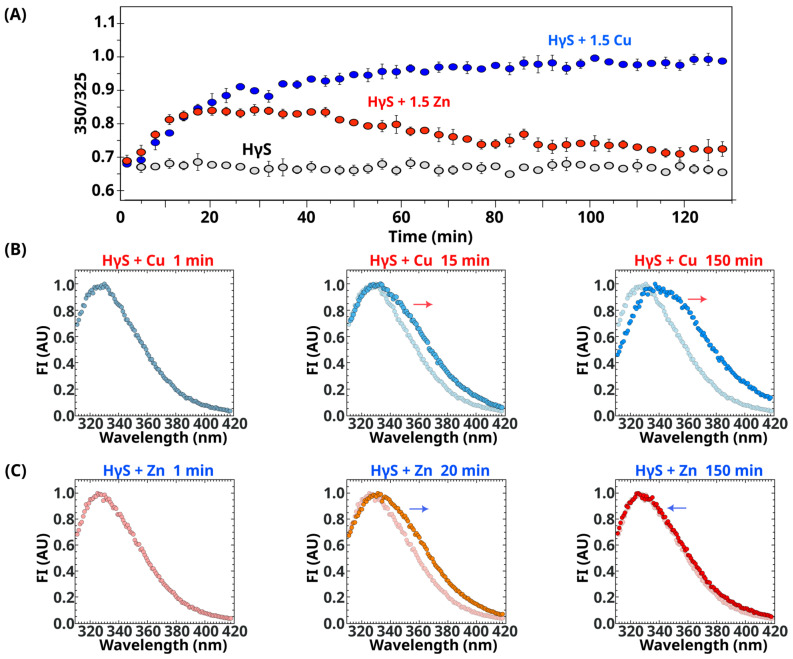
Effect of Cu(II) and Zn(II) ions on the folding of HγS crystallin. (**A**) Fluorescence intensity ratio 350/325 as a function of time at 37 °C of HγS crystallin in the absence (gray), presence of Cu(II) (blue) and Zn(II) (red). (**B**) Normalized fluorescence spectra over time of HγS crystallin in the presence of 1.5 equivalent ratios of Cu(II). A shift to the right (indicated with an arrow) can be observed, indicating the exposure of tryptophan residues to the solvent. (**C**) Normalized fluorescence spectra over time of HγS crystallin in the presence of 1.5 equivalent ratios of Zn(II). First, a shift to the right can be observed, indicating the exposure of tryptophan residues to the solvent; then, a shift to the left indicates the burial of tryptophan residues. The direction of the shifts is indicated with arrows.

In the absence of metal ions, the ratios remained constant during the experiment (Figure 4A gray line). In the presence of 1.5 equivalent ratios of Cu(II), HγS crystallin increased the 350/325 ratio during the first hour, and subsequently, the value of the ratio remained close to 1, which indicates that the protein underwent conformational changes in the presence of this metal ion. At the end of the assay, the emission maximum was 337 nm, suggesting that the protein was partially unfolded, with some tryptophan residues exposed to the solvent (Figure 4).

In the presence of Zn(II), the behavior of the ratio of 350/325 for HγS crystallin was different. In the first few minutes, the 350/325 ratio increased, indicating that some of the buried tryptophan residues experienced some exposure to the solvent. Nevertheless, a decrease in the ratio was observed after this time, returning to values similar to those shown by the folded protein in the absence of the metal ion (Figure 4A).

Figure 4B shows the emission spectra at different times in the presence of metals. For Cu(II), the emission maximum shifts to the right with time until it reaches 337 nm, but for Zn(II), the emission maximum shifts to the right in the first few minutes; however, later, the maximum begins to return to the initial value. At the end of the experiment, the spectrum is similar to the initial spectrum (Figure 4C). This finding indicates that in the Zn(II)-induced process, tryptophan residues are exposed to the solvent in the first step and then reburied during the formation of the final aggregate.

### 3.3. Characterization of the HγS Crystallin-Metal Ions by NMR Spectroscopy

To detect residue-specific changes induced by Zn(II) or Cu(II) binding, we used NMR spectroscopy. Two-dimensional ^1^H-^15^N correlation spectra of HγS crystallin showed well-dispersed resonances at 37 °C, consistent with the existing backbone resonance assignments deposited in the BioMagResBank [30,31]. However, not all the expected amide signals were observed, as the spectrum contained only 143 of the 169 expected resonances (excluding the prolines). The absence of these signals was most likely due to intermediate exchange broadening.

Changes in the chemical environments of the atoms upon metal binding would result in chemical shift changes in the NMR spectra. To analyze the effects of the interaction of HγS crystallin with Cu(II), we analyzed the decrease in signal intensity due to the copper paramagnetic effect.

**Figure 5:**
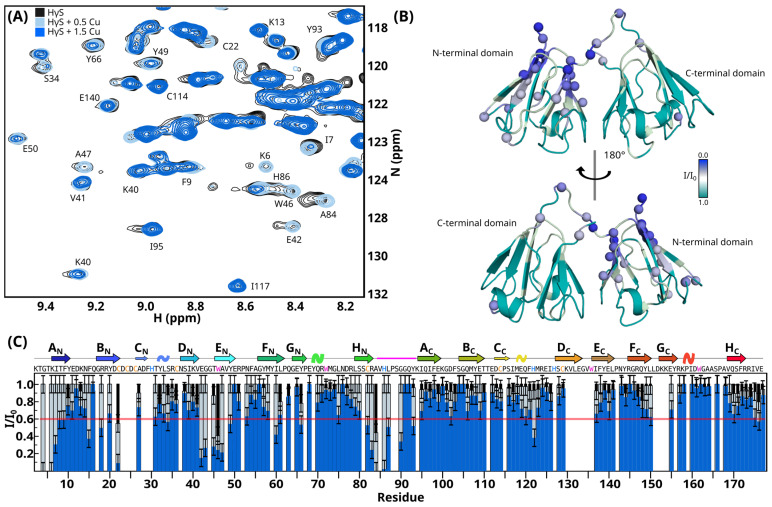
Characterization of the HγS crystallin—Cu(II) interaction by NMR. (**A**) Overlay of a region of ^1^H-^15^N HSQC spectrum of free HγS crystallin (black) and HγS crystallin in the presence of 0.5 (light blue) and 1.5 equivalent ratios of Cu(II) (blue). (**B**) Cu(II)-induced weighted intensity changes (I/I_0_) are mapped onto the HγS crystallin structure, color-coded via a linear ramp from cyan (no change) to blue (maximal signal loss). The residues for which the effect of copper binding could not be determined are shown in light gray. (**C**) Induced weighted intensity changes (I/I_0_) induced by the binding of Cu(II) per residue.

Unexpectedly, with 0.5 and 1.5 equivalent ratios of Cu(II), the signal obtained from the spectrum was lower, presumably due to the formation of large aggregates that were not observed in NMR. To compensate for this widespread loss of signal, we normalized the intensity to an internal signal before analysis. We detected a specific signal loss for residues 23, 50, 84, and 86 at 0.5 eq. When the analysis was performed for 1.5 eq., loss of signals was observed in the N-terminal residues, B_N_ strand and loop B_N_-C_N_, loop D_N_-H_N_, the linker regions and residues in the C_C_-D_C_ loop (Figure 5 and Figure S4).

**Figure 6:**
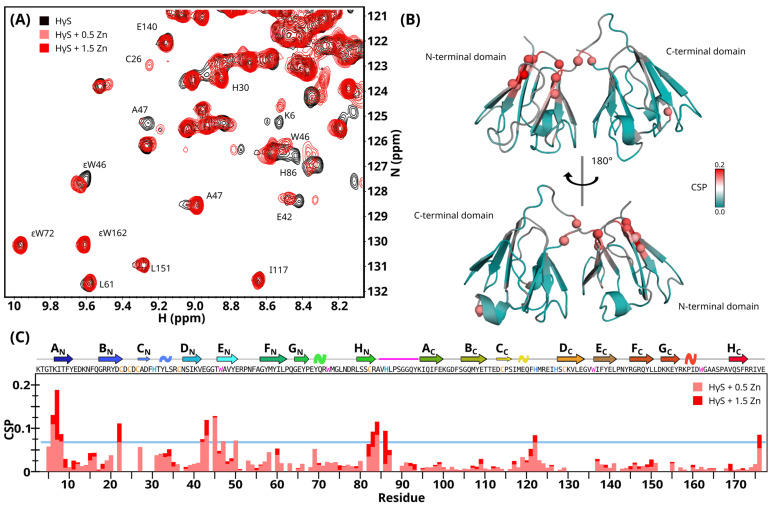
Characterization of the HγS crystallin-Zn(II) interaction by NMR. (**A**) Overlay of a region of the ^1^H-^15^N HSQC spectrum of free HγS crystallin (black) and HγS crystallin in the presence of 0.5 (light red) and 1.5 equivalent ratios of Zn(II) (red). (**B**) Zn(II)-induced chemical shift perturbations (CSPs) are mapped onto the HγS crystallin structure, color-coded via a linear ramp from cyan (no change) to red (maximal signal loss). The residues for which the effect of zinc-binding could not be determined are shown in light gray. (**C**) Chemical shift perturbations induced by the binding of Zn(II) per residue.

With 0.5 and 1.5 equivalent ratios of Zn(II), the NMR spectra revealed modest but site-specific chemical shift perturbations constrained to the N-terminal domain. We observed CSP in the N-terminal residues, B_N_ strand and loop B_N_-C_N_, E_N_ strand, F_N_ strand and H_N_ strand, C_C_- D_C_ loop, and C-terminal (Figure 6). Normalized weighted average ^1^H and ^15^N shift differences were color-mapped onto the structure to identify the possible metal-binding regions of the protein (Figure 6B).

Overall, similar regions of HγS crystallin were perturbed by the binding of Cu(II) or Zn(II) to the protein (Figure 5B and Figure 6B). The residues most affected by the presence of metal ions are located in the N-terminal domain and in the linker loop, which confirms that the interaction sites of Cu(II) and Zn(II) ions are located in these regions. But we also observed small changes in the C-terminal domain (Figure S5) and in other regions that have not been described as being involved in metal bonding. Interestingly, for both metals, when we looked at the side chain signal of Trp residues, we detected substantial changes only for W46, while W72, W136, and W162 remain unperturbed (Figure S6). These data suggest that the changes observed in florescence are mainly due to changes in W46 and not the second Trp in the N-terminal domain nor the C-terminal domain Trps.

## 4. Discussion

Our results show that, indeed, 1.5 equivalent ratios of Cu(II) or Zn(II) induce aggregation of the HγS crystallin solution over time at 37 °C. Although both metal ions induce the formation of high-molecular-weight light-scattering aggregates, the aggregation pathways for each metal are different.

The binding of copper promotes several sequential processes, as observed by ITC, which include partial unfolding and aggregation. Similar effects have been observed with HγD; however, copper with this homologous protein resulted in a delay of minutes in the formation of large aggregates due to the time that copper partially unfolded the protein [22]. The HγS crystallin data revealed the formation of large aggregates immediately after metal addition (Figure 2); However, Trp fluorescence shows a gradual increase in the ratios of I350/I325, suggesting that aggregates with folded proteins are first formed by metal bridges and then over time the N-terminal domain partially unfold.

Previous studies with HγS crystallin suggested that Cu(II) interacts with cysteines C22, C24, or C26, which are accessible to solvents, and that C82 participates in the formation of a disulfide bridge [25]. Our NMR chemical shift data show that residues 80–90 and residues in the vicinity of cysteines 22 and 26 are affected by the presence of Cu(II), consistent with the proposed interactions. We also detected perturbation in the C-terminal domain, specifically around the loop connecting the CC and DC strands, suggesting that copper may also bind in this area. The extent to which each copper binding site is responsible for aggregation remains to be determined.

Another study suggested that C129 in HγS crystallin could form disulfide bridges with C114 due to interaction with metal ions [26]; however, our NMR data do not support this at low metal concentrations.

It has been proposed that Cu(II) induces an intermediate where the N-terminal domain is completely unfolded; however, NMR and fluorescence data do not support this interpretation. Only the side chain of W46 is affected for copper, where we observe signal loss due to the paramagnetic ion, while W72 and its surroundings do not suffer any change (Figure S6), neither signal loss nor change in position, which suggests that a partial unfolding of the N-terminal domain. Interestingly, the final protein aggregate does not completely bury all tryptophan residues, as observed by fluorescence.

In the case of zinc, previous biochemical data suggested that Zn(II) did not promote protein aggregation [27]. However, a report later [28] and the data presented here are consistent with the ability of Zn(II) to induce aggregation in solution.

Zn(II) preferentially interacts with histidine or cysteine residues, forming coordination complexes involving Zn(II) and at least three amino acid side chains (usually His, Cys, Asp, and/or Glu) [35,36]. The HγS crystallin protein contains four histidine residues (H30, H86, H122, and H127) and seven cysteine residues (C22, C24, C26, C36, C82, C114, and C129); their distribution is shown in Figure 1.

It could be proposed that the coordination complex that binds Zn(II) is formed with residues from two HγS crystallin molecules. In this complex, H86 or H122 from one protein and a Cys of the C23-C25-C27 triad of another protein could be involved since the side chains of these residues are exposed and oriented toward the solvent. The formation of this complex could also explain the perturbation of residues spatially close to these regions. This mechanism of aggregation aligns with the proposal that Zn(II) induces aggregation through cross-linking in a cysteine-dependent manner [28]. Other residues affected by the presence of Zn(II) include W46 and residues located at the interface of the two domains. This perturbation could be attributed to small conformational changes occurring in the domain during interaction with Zn(II).

Zn(II) binding also promotes several events in the protein. We observed a small change in fluorescence, indicating some exposure of the buried tryptophan residues after metal binding. This increase in the maximum fluorescence ratio of 350/325 is followed by a decrease in values similar to the initial ones, indicating that the tryptophan residues change to a buried state. This return does not necessarily mean that the protein returns to the same conformational state but rather that the environment of W46 appears to be buried again in the aggregate. Given the flexibility of the linker between the domains and the paucity of contacts between them, we might be detecting an opening between the domains, which could promote aggregation and easily rebury the entire interface during aggregation.

Interestingly, for 0.5 equivalents for both metals, we observed changes in the NMR data, which could imply that even this low concentration could promote some changes leading to aggregation, although probably on a longer time scale than proved in this study. 

## 5. Conclusions

The results obtained demonstrate that copper and zinc interact with HγS crystallin and induce opacification over time at 37 °C even at low equimolar ratios. The features of HγS crystalline interacting with metal ions presented here provide insights into the early steps of the aggregation process not described before. While both metals induce aggregation through the formation of metal-bridged species, the interaction of HγS crystallin with Cu(II) first promotes the formation of metal bridges but also induces partial unfolding of proteins, leading to the formation of aggregates in which at least one tryptophan residue is exposed to the solvent. In contrast, Zn(II) interactions result in a structural rearrangement of the protein, causing a temporary exposure of a tryptophan residue to the solvent. Once the aggregates form, the tryptophan residues return to a chemical environment where they become buried again.

## Data Availability

Data is contained within the article or supplementary material.

## Supplementary Materials

The following supporting information can be downloaded at: https://www.mdpi.com/article/10.3390/biom14121644/s1, Figure S1: Effect of metal ions on the aggregation of HγS by DLS; Figure S2: Metal binding by ITC; Figure S3: Effect of Zn(II) and Cu(II) on the thermal stability of HγS; Figure S4: HSQC HγS – Cu(II); Figure S5: C-terminal residues interaction by NMR; Figure S6: Characterization of the TRP interaction by NMR; Figure S7: SDS-PAGE of the purified protein HγS.
