# Supplementary material for: Characterization of the Interaction of Human γS Crystallin with Metal Ions and Its Effect on Protein Aggregation
Source: Biomolecules. 2024 Dec 21;14(12):1644. doi: 10.3390/biom14121644 (PMC11674332; doi:10.3390/biom14121644)
Supplement: Supplementary file 1 [file biomolecules-14-01644-s001.zip › biomolecules-3322058-supplementary.pdf]

Supplementary Materials

Characterization of the Interaction of Human γS Crystallin with Metal Ions and Its Effect on Protein Aggregation

Reinier Cardenas [1], Arline Fernandez-Silva [1], Vanesa Ramirez-Bello [1,2], and Carlos Amero [1,*]

[1] LABRMN, Centro de Investigaciones Químicas, Instituto de Investigación en Ciencias Básicas y Aplicadas, Universidad Autónoma del Estado de Morelos, Morelos 62209, Mexico; reinier.cardenazmen@uaem.edu.mx (R.C.); arline.fernandezsil@uaem.edu.mx (A.F.-S.); vanesa.ramirezbel@uaem.edu.mx (V.R.-B.)

[2] Grupo de Investigación en Producción y Sanidad en Ciencias Veterinarias y Zootecnias (PROSAVEZ),
Facultad de Medicina Veterinaria y Zootecnia, Fundación Universitaria San Martín, Cali 760001, Colombia

* Correspondence: carlosamero@uaem.mx; Tel.: +52-777-329-7900 (ext. 6043)

Figure S1: Effect of metal ions on the aggregation of HγS by DLS.
Figure S2: Metal binding by ITC.
Figure S3: Effect of Zn(II) and Cu(II) on the thermal stability of HγS.
Figure S4: HSQC HγS – Cu(II).
Figure S5: C-terminal residues interaction by NMR.
Figure S6: Characterization of the TRP interaction by NMR.
Figure S7: SDS-PAGE of the purified protein HγS.

Academic Editor: Tzanko I. Doukov

Received: 3 November 2024
Revised: 11 December 2024
Accepted: 18 December 2024
Published: 21 December 2024

Citation: Cardenas, R.; Fernandez-Silva, A.; Ramirez-Bello, V.; Amero, C. Characterization of the Interaction of Human γS Crystallin with Metal Ions and Its Effect on Protein Aggregation. *Biomolecules* **2024**, *15*, x. https://doi.org/10.3390/biom14121644

Copyright: © 2024 by the authors. Submitted for possible open access publication under the terms and conditions of the Creative Commons Attribution (CC BY) license (https://creativecommons.org/licenses/by/4.0/).

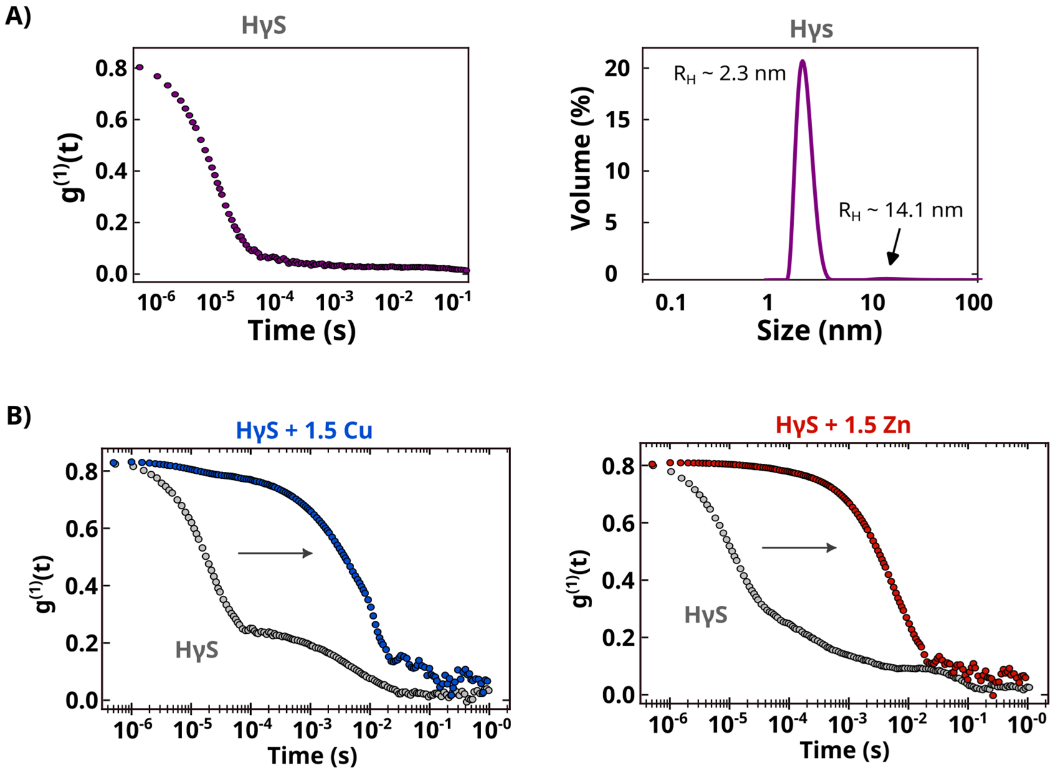

Figure S1: Effect of metal ions on the aggregation of HγS by DLS. A) DLS correlograms of HγS and corresponding size distribution diagram. B) DLS correlograms of HγS crystallin in the absence (gray) and presence of Cu(II) (blue). DLS correlograms of HγS crystallin in the absence (gray) and presence of Zn(II) (red). A shift to the right can be observed, indicating the formation of large aggregates.

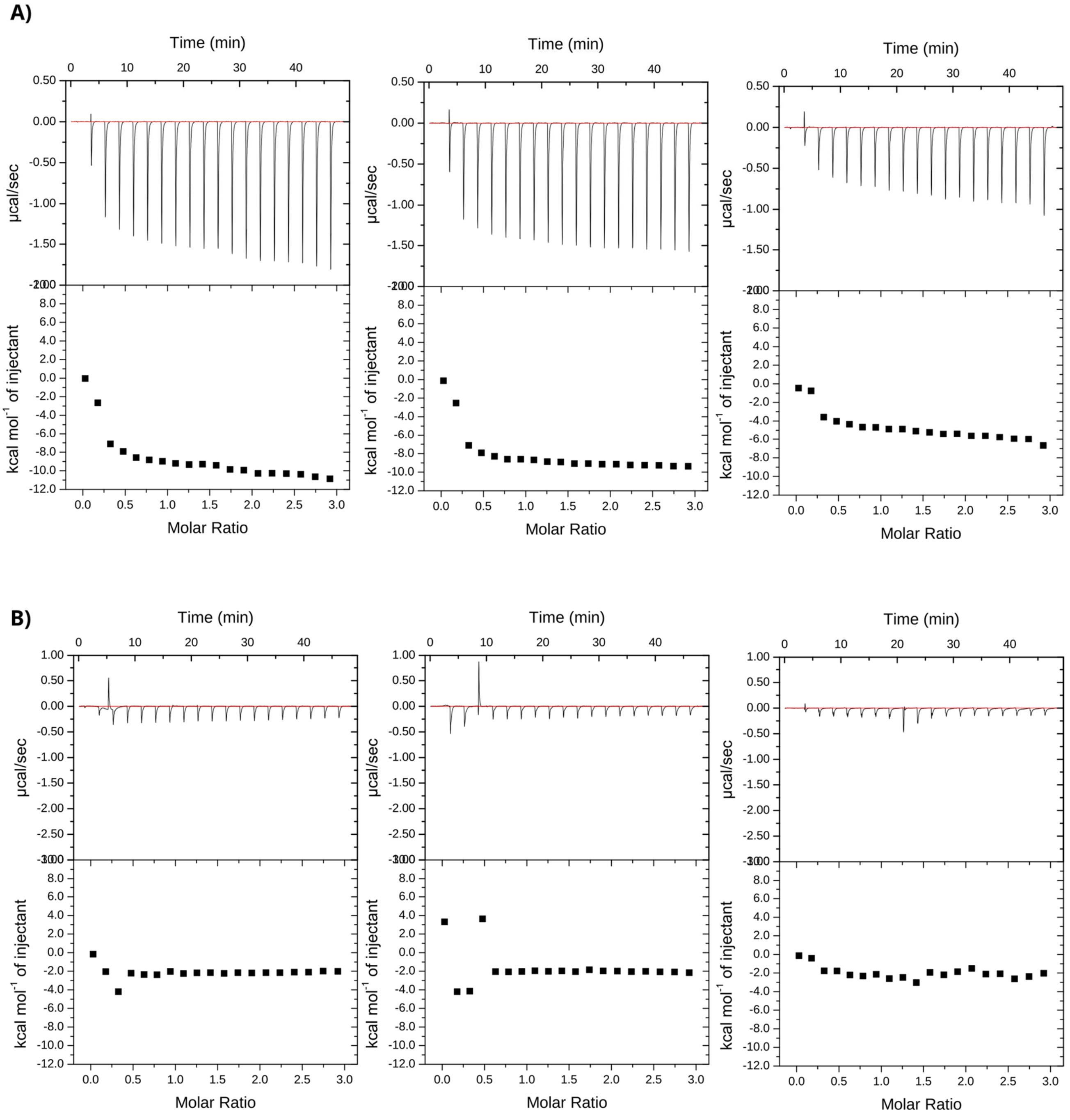

Figure S2: Metal binding by ITC. A) Isothermal titration calorimetry of HγS bound to Cu(II). B) Isothermal titration calorimetry of HγS bound to Zn(II). The top shows the experimental isothermal titrations, whereas the bottom shows the reaction heat. Both metals exhibited complex behavior involving several processes accounting for the heat reactions.

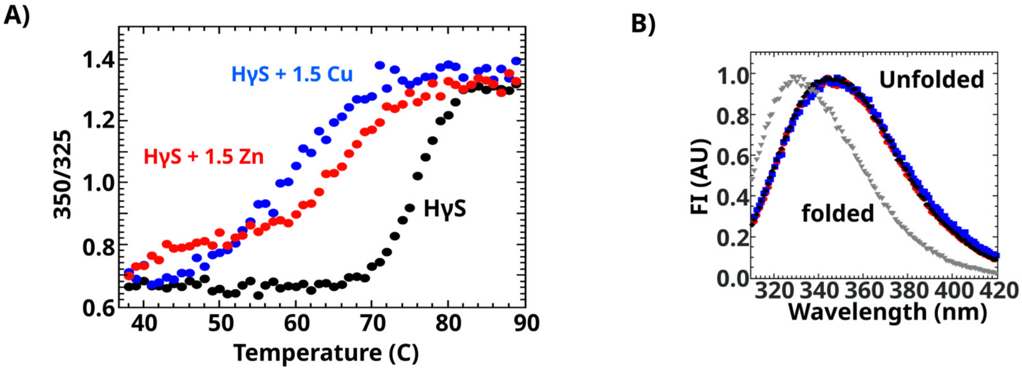

Figure S3: Effect of Zn(II) and Cu(II) on the thermal stability of HγS. A) Thermal unfolding of HγS in the absence (black) and in the presence of 1.5 equivalents of Cu(II) (blue) and Zn(II) (red) B) Normalized Fluorescence Spectra of HγS before and after the assay. In the absence of metal ions, the apparent Tm is 75.7 °C. In the presence of metal ions, apparent Tm decreases to 65.3 °C for Zn(II) and to 59.1 °C in the presence of Cu(II). It was observed that the emission maximum shifted from 325 nm to 350 nm, both in the absence and presence of the metal ions.

The temperature was increased from 37 °C to 90 °C, and the fluorescence intensity at 350 nm and 325 nm was measured at each temperature. Subsequently, the ratio of the fluorescence intensity between 350/325 nm was calculated. Once the fluorescence intensity ratio was calculated for each temperature, the experimental data were fitted to a Boltzmann sigmoid curve.

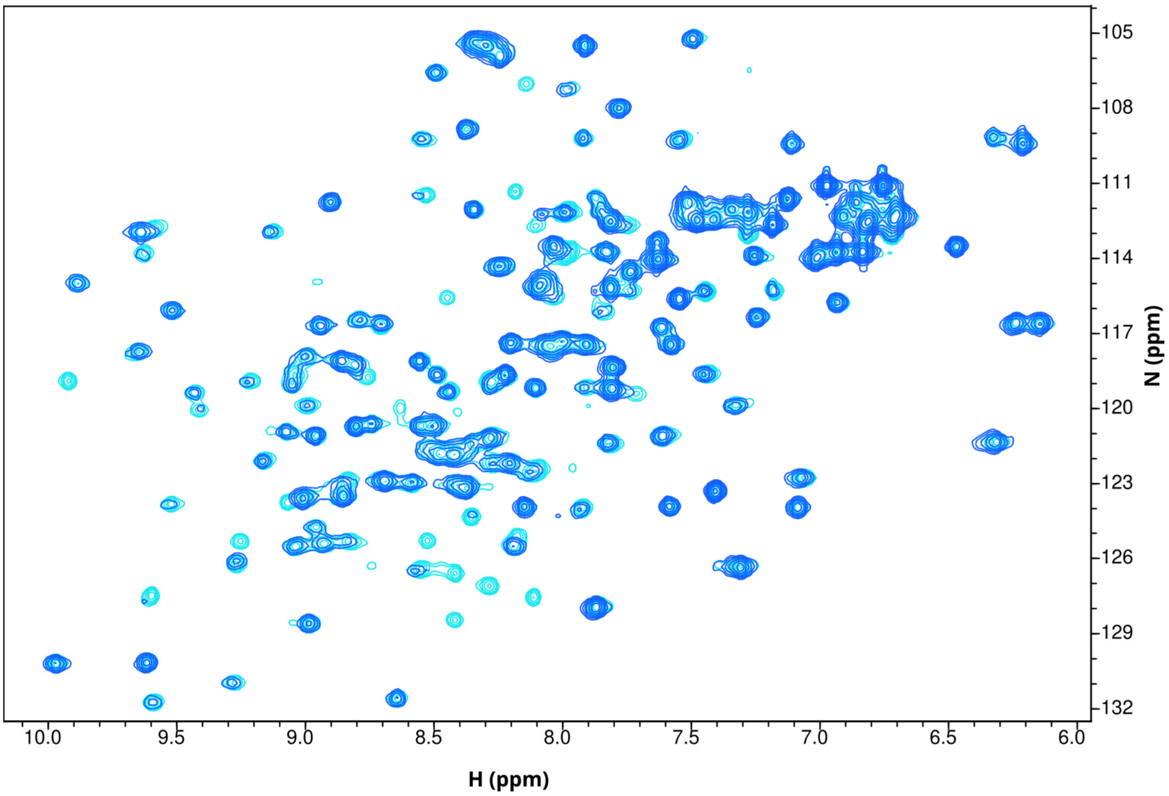

Figure S4: HSQC HγS – Cu(II). A) ^{1}H-^{15}N HSQC spectrum of HγS in the presence of 0.5 (cyan) and 1.5 equivalents of Cu(II) (blue).

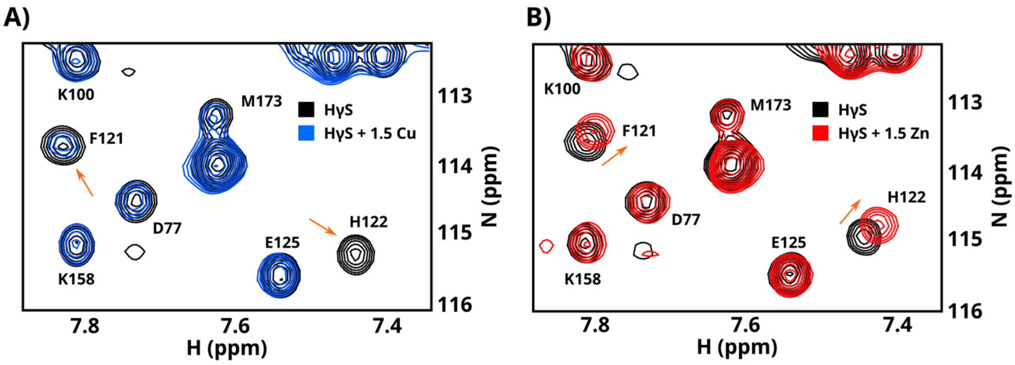

Figure S5: C-terminal residues interaction by NMR. A) Overlay of a region of the ^{1}H-^{15}N HSQC spectrum of free HγS (black) and HγS in the presence of 1.5 equivalents of Cu(II) (blue). B) Overlay of a region of the ^{1}H-^{15}N HSQC spectrum of free HγS (black) and HγS in the presence of 1.5 equivalents of Zn(II) (red). Changes in peak intensity and position for residues F121 and H122 are shown.

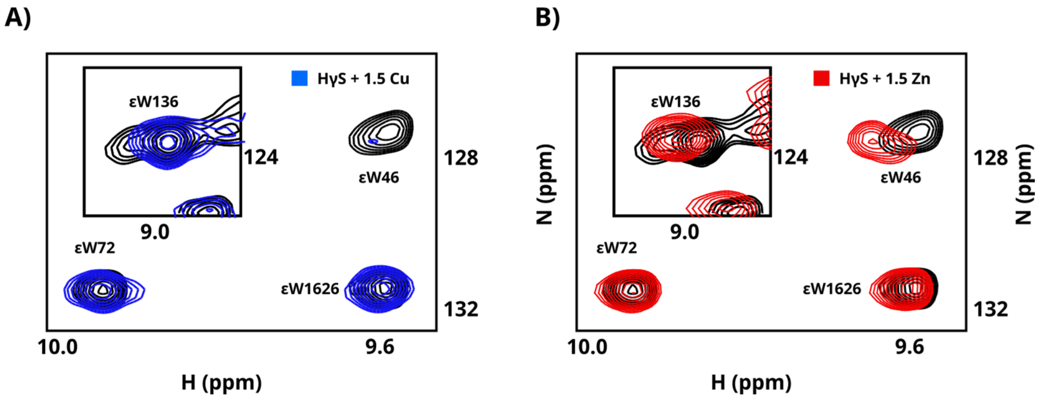

Figure S6: Characterization of the TRP interaction by NMR. A) Overlay of a Trp side chain signals region of the ^{1}H-^{15}N HSQC spectrum of free HγS (black) and HγS in the presence of 1.5 equivalents of Cu(II) (blue). B) Overlay of a Trp side chain signals region of the ^{1}H-^{15}N HSQC spectrum of free HγS (black) and HγS in the presence of 1.5 equivalents of Zn(II) (red).

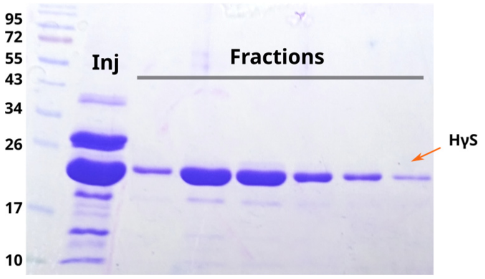

Figure S7: SDS-PAGE of the purified protein HγS. Coomassie-stained SDS polyacrylamide gel analyzing the purification of the protein is shown. Lane 1: Molecular weight marker. Lanes 2: Sample injected. Line 3 – 8: Fractions.
